# Mechanisms of Direct and Indirect Presentation of Self-Antigens in the Thymus

**DOI:** 10.3389/fimmu.2022.926625

**Published:** 2022-06-14

**Authors:** Jiří Březina, Matouš Vobořil, Dominik Filipp

**Affiliations:** Laboratory of Immunobiology, Institute of Molecular Genetics of the Czech Academy of Sciences, Prague, Czechia

**Keywords:** thymus, central tolerance, antigen presentation, thymic epithelial cells, dendritic cells, cooperative antigen transfer

## Abstract

The inevitability of evolution of the adaptive immune system with its mechanism of randomly rearranging segments of the T cell receptor (TCR) gene is the generation of self-reactive clones. For the sake of prevention of autoimmunity, these clones must be eliminated from the pool of circulating T cells. This process occurs largely in the thymic medulla where the strength of affinity between TCR and self-peptide MHC complexes is the factor determining thymocyte fate. Thus, the display of self-antigens in the thymus by thymic antigen presenting cells, which are comprised of medullary thymic epithelial (mTECs) and dendritic cells (DCs), is fundamental for the establishment of T cell central tolerance. Whereas mTECs produce and present antigens in a direct, self-autonomous manner, thymic DCs can acquire these mTEC-derived antigens by cooperative antigen transfer (CAT), and thus present them indirectly. While the basic characteristics for both direct and indirect presentation of self-antigens are currently known, recent reports that describe the heterogeneity of mTEC and DC subsets, their presentation capacity, and the potentially non-redundant roles in T cell selection processes represents another level of complexity which we are attempting to unravel. In this review, we underscore the seminal studies relevant to these topics with an emphasis on new observations pertinent to the mechanism of CAT and its cellular trajectories underpinning the preferential distribution of thymic epithelial cell-derived self-antigens to specific subsets of DC. Identification of molecular determinants which control CAT would significantly advance our understanding of how the cellularly targeted presentation of thymic self-antigens is functionally coupled to the T cell selection process.

## Introduction

The immune system is considered to be one of the most complex entities in the body. It generates various specialized cells which primarily detect and eliminate pathogens to protect the host. This process of immune “self-nonself discrimination” is a fundamental attribute of a healthy immune system (1). Since T cell antigen receptors (TCRs) are generated by random somatic recombination without regards to a target, i.e. self or nonself-specific, T cells that express a potentially dangerous self-reactive TCR are either removed through the process of negative selection (recessive tolerance) or diverted into thymic regulatory T cells (Tregs), the lineage of cells with the propensity to downregulate inflammatory responses (dominant tolerance) (2–4). These processes, which together are generally classified as central tolerance, are operational in the thymus and robustly limit the self-reactive repertoire within the T cell population (5, 6). One of the key molecules of central tolerance is the Autoimmune regulator (AIRE). AIRE has been determined to be a transcriptional regulator that promotes the “promiscuous”, or “ectopic” expression of thousands of tissue-restricted self-antigens (TRAs), specifically in medullary thymic epithelial cells (mTECs) (7).

A critical part of the processes associated with central tolerance occurs in the thymic medulla and depends on the presence of various types of dendritic cells (DCs), B cells, and highly specialized non-hematopoietic antigen presenting cells (APCs) known as mTECs. These cells participate in recessive and dominant tolerance *via* cell autonomous antigen presentation (6). Recent data also suggests that cooperation between these cells in an unidirectional antigen transfer i.e., mTECs to DCs, which we will refer to as cooperative antigen transfer (CAT), is required for the efficient induction of T cell tolerance and Treg selection (8, 9). However, while CAT represents an important physiological pathway for imposing T cell tolerance, until recently, it was unclear how many cell subsets of mTECs and DCs participate in this process. In addition, it was not known how distinct DC subsets are recruited to mTECs resulting in efficient CAT, whether these cells interact in a stochastic or deterministic fashion and perhaps the most importantly, the specific roles of these cells in the establishment of tolerance.

In this review, we will highlight the current knowledge concerning the pathways by which self-antigens are presented in the thymus and how they lead to establishment of both recessive and dominant tolerance. We will also examine and discuss the possible molecular mechanisms underpinning CAT. Finally, we will draw attention to the current model of CAT which proposes distinct preferences of DC subsets in the acquisition of thymic epithelial cell-derived antigens.

## Direct Antigen Presentation

The mechanisms of central tolerance are based on the premise that developing thymocytes (either CD4^+^ or CD8^+^) gauge their level of autoreactivity *via* the interactions of their TCRs with self-peptide-MHC (pMHC) complexes presented on the surface of thymic APCs (6). However, it is quite striking how developing thymocytes are able to see an entire collection of host self-antigens in an anatomically confined thymic space remained enigmatic over a long period of time. In the late 1980’s, researchers unexpectedly found that some cell types were able to express seemingly irrelevant tissue specific genes (10, 11). This phenomenon was referred to as “ectopic gene expression” and led to the proposal that thymus cells can create a “patchwork quilt” of self-antigens which they present to developing T cells (12). These self-antigens are classified into three main groups: (i) antigens that exhibit a ubiquitous expression pattern; (ii) antigens which are specifically expressed by particular cell subtypes under certain conditions (such as those expressed by class-switched B cells); and (iii) antigens whose expression is limited to only one or up to a few anatomical places outside the thymus (7, 13–15). The latter category of self-antigens represent tissue-restricted antigens (TRAs) whose expression has been attributed to a rare population of thymic stromal cells, mTECs (13). The specifics of TRAs expression are very different from those of standard gene expression in peripheral tissues: (i) TRAs, whose production is tightly regulated, are expressed by a single mTEC in a stochastic manner (only 1-3% of all mTECs express a given TRA at any given time) (16, 17); (ii) TRA genes are often expressed from a single-allele using alternative transcriptional start sites (18); (iii) sex-related genes are expressed by mTECs irrespective of gender (16, 19); (iv) TRAs contain several development-related genes that are expressed by mTECs with no connections to the developmental status of the organism (13). These attributes enable mTECs to express a broad repertoire of self-antigens that are needed for proper T cell selection.

Recently, RNA sequencing technology has helped determine that mTECs express more than 18,000 genes, which represent approximately 85% of the protein-coding genome (20, 21). Compared to other cell types from different tissues, the number of genes typically range from 12,000 to 14,000 (i.e., 60- 65% of coding genome) (22). Remarkably, there are approximately 4,000 genes in mTECs regulated by AIRE (7, 21). Thus, a set of mTEC-dependent TRAs can be expressed in an AIRE-dependent or AIRE-independent manner. While the regulation of AIRE-independent promiscuous gene expression is still not completely understood, the transcription factor Family Zinc Finger 2 (FEZF2) was suggested to play a complementary role in mediating immune tolerance to AIRE-independent TRAs (23). Also, as mentioned above, any TRA at any given time is expressed only by 1-3% of mTECs and one mTEC is able to co-express approximately 100-300 TRAs (16, 17, 24). Correspondingly, it was postulated that 200-500 mTECs are sufficient to cover the entire TRA repertoire (22). This suggests that TRA expression is in the thymus controlled by the rules of “ordered stochasticity”, where the initial co-expression pattern of TRAs is stochastic, but then is highly regulated by a coordinated set of events (24).

The previously mentioned process, in which the recognition of epitopes derived from TRAs by self-reactive T cells leads to their deletion or conversion to Treg-lineage was described in classical studies that employed neo-self-antigen technology. Using mouse models in which the expression of hen egg lysozyme (HEL) or membrane-bound chicken ovalbumin (mOVA) was driven by the rat insulin promotor (RIP), and thus expressed in an AIRE-dependent manner, it was described that *Aire* knockout (KO) mice possessed an increased number of neo-self-antigen specific (TCR-HEL or OT-II, respectively) CD4^+^ T cells, suggesting a role of AIRE^+^ mTECs in clonal deletion (25, 26). Also, using tetramer enrichment technology, it was shown that polyclonal T cells which are specific for particular TRAs are modestly increased in *Aire* KO mice (14).

Using mTEC-specific neo-self-antigen models along with TCR transgenic systems, *Aschenbrenner et al.* suggested that AIRE-expressing mTECs also play a crucial role in Tregs generation (27). This was confirmed for organ specific Tregs which required AIRE-dependent expression of TRAs as well (19, 28). The importance of AIRE itself in shaping the Tregs repertoire was implied by deep sequencing of the complete TCRα genes in Tregs and conventional T cells (Tconv) that were isolated from *Aire* KO mice. This experiment showed that in the absence of AIRE, TCR sequences which were usually found among a Treg lineage could be detected in the repertoire of Tconv cells (29). However, other studies presented evidence that AIRE is essential for the generation of Tregs, mostly during the neonatal period of life (30–32).

Even though the mechanisms of central tolerance have been extensively studied, there is still a paucity of information detailing the mechanism controlling the decision-making process between clonal deletion and Tregs generation. The simplest models used to illustrate the specifics of the mechanism have been based on the fact that high-affinity interaction leads to clonal deletion, while weaker interactions have resulted in Tregs generation (6). This is in agreement with studies that have used T cell transgenic systems specific to neo-self-antigens which, however, exhibit a high affinity for TCR-pMHC interaction and are skewed to massive clonal deletion rather than Tregs deviation (25, 26). On the other hand, the MHC-tetramer technology which operates using natural TCR affinities provides evidence that the clonal deletion of TRA-specific thymocytes is far from being complete and is rather biased towards Treg selection (14, 33–35). Specifically, this phenomenon was described using MHCII tetramers specific to neo-self-antigens, whose expression is restricted to either all (ubiquitous antigens) or various tissues (TRA-like expression pattern). It was shown that the recognition of ubiquitous antigens led to a massive deletion of antigen-specific T cells, whereas the recognition of TRA-like antigens predominantly promoted the diversion to Treg lineage (14, 34). This observation opened the question of whether different types of APCs play a non-redundant role in mediating clonal deletion or Tregs selection.

It has been known for more than a decade that the thymic population of APCs is heterogeneous in its nature since it includes cells of hematopoietic and non-hematopoietic origin. In recent years, the robustness of single-cell RNA sequencing (scRNAseq) has not only yielded a vast amount of information about thymic APC heterogeneity (36–40) but have provided a set of new markers to distinguish these APC subsets. Historically, thymic epithelial cells (TECs) have been divided into two major populations: mTECs and cortical TECs (cTECs) (41). Recently, combining lineage tracing technology with scRNAseq, it was revealed that mTECs are highly heterogeneous and comprise of multiple populations that have different molecular and functional characteristics. These include immature and CCL21^+^ mature mTEC^Low^, AIRE^+^ mTEC^High^, corneocyte-like mTECs (Post-Aire mTECs), and tuft cell-like mTECs (36, 42–44). Even though there are publications describing the roles of particular mTEC-subtypes, such as the attraction of single positive (SP) thymocytes to the medulla (CCL21^+^ mTEC^Low^) (45), modulation of Type 2 immune responses (tuft cell-like mTECs) (36, 46), or production of pro-inflammatory cytokines (Post-Aire mTECs) (47), the exact function of specific TEC-subtypes in mechanisms of clonal deletion or Tregs selection is largely unknown. The data which has been compiled so far suggests that the AIRE^+^ mTEC^High^ subset, by presenting peptides derived from TRAs, plays a non-redundant role in Tregs generation, whereas the other mTEC-subpopulations predominantly participate in clonal deletion due to the presentation of ubiquitous antigens (14, 34, 35). Because the direct MHC-dependent interaction between developing T cells and mTECs is required for proper medullary organization, assessing the exact function of TEC-subtypes in tolerance would require the development of models that target MHC expression in particular TECs subpopulations. So far, this aim was partially achieved with AIRE^+^ mTECs where the MHCII transactivator, C2TA, was knocked down by *Aire* promotor-driven shRNA. C2TAkd mice showed a moderate increase in CD4^+^ T cells suggesting the role of mTECs in clonal deletion. Interestingly, the introduction of MHCII deficient bone marrow to this system further increased the number of CD4^+^ T cells suggesting that mTECs and DCs play non-redundant roles in clonal deletion (48). Further analysis comparing the unique TCRα sequences of CD4^+^ T cells from C2TAkd and mice with MHCII deficient bone marrow revealed that even though mTECs were able to perform clonal deletion, their relative contribution to this process was minimal compared to bone marrow-derived DCs (8). Since such a non-redundant role of mTECs and DCs has also been shown for Tregs selection, it indicated the functional dichotomy of epithelial and DC cellular networks involved in the establishment of central tolerance.

Historically three major conventional subtypes of DC have been described within the thymus: plasmacytoid DCs (pDC), classical DC Type 1 (cDC1), and classical DC Type 2 (cDC2) (49). These major DC subtypes, commonly expressing CD11c marker, are delineated by their expression of lineage specific surface markers and transcription factors. The cDC1 population is defined by the expression of the chemokine receptor, XCR1, and requires the transcription factors BATF3 and IRF8 (50), whereas cDC2 subset expresses SIRPα and partially requires the transcription factor IRF4 (51). In general, the function of DCs in central tolerance was first determined in CD11c-Cre-DTA mice in which their genetic ablation led to impaired clonal deletion of T cells and development of severe autoimmunity (52), demonstrating their indispensable role in establishment of tolerance. Along with the conventional DC-subtypes mentioned above, the thymus also accommodates other DC-like subsets, such as monocyte-derived DCs (moDC) or cells that resemble activated or migratory DCs which are present in peripheral lymphoid and non-lymphoid tissues (53–55). These cells are characterized by increased expression of CCR7 and have been described as activated DCs (aDC) (39, 54).

The function of cDC1 has been mostly attributed to the cross-presentation of mTEC-derived self-antigens to developing T cells (described in detail in the next chapter). Also, the previously mentioned CCR7^+^ aDC derived from XCR1^+^ cDC1 have been shown to be particularly important in this process (54). On the other hand, the activated DCs also change their displayed self-peptidome, through changes in proteasome subunits, phagosome enzymes, and autophagy proteins. Thus, aDC have the potential to tolerize developing T cells to self-antigens that are associated with their activation (56). This should be particularly important during inflammation in the immune periphery when DCs are activated to protect the host from the development of autoimmune reactions towards self-molecules that are associated with DC-activation (54). In contrast to cDC1, cDC2 has been shown to originate in the periphery, and thus capable of presenting antigens acquired in peripheral tissues (57). This was first postulated by *Bonasio et al.* showing that OT-II thymocytes were selectively deleted in the thymus after intravenous injection of OVA-loaded exogenous DCs (58). More recently, the specific population of trans-endothelial DCs was described to be responsible for delivering and presenting peripheral blood-borne antigens to the thymus for clonal deletion (59). Interestingly, the positioning and function of these cells was shown to be dependent on CX3CR1 expression which also marks the specific population of DCs previously associated with presentation of intestinal-derived microbial antigens in the thymus (60). This data suggests that CX3CR1^+^ cDC2 cells are responsible for the delivery of peripheral antigens to the thymus for T cell clonal deletion (53, 59, 60). In addition, the thymic population of pDC was also shown to be involved in mediating central tolerance since adoptively transferred OVA-loaded pDCs migrated to the thymus and promoted the deletion of OT-II thymocytes. Interestingly, the migration of pDC into the thymus was shown to be dependent on the CCR9/CCL25 axis, which is also important for migration of cells into intestinal tissues (61). This suggests that in addition to cDC2, pDCs could also be responsible for presentation of peripheral antigens in the thymus.

The thymus also accommodates a population of B cells that seem to be “licensed” for antigen presentation, the phenomenon in which the thymic microenvironment plays an indispensable role (62, 63). The thymus contains a population of class-switched B cells that express AIRE and thus can present some of the AIRE-dependent antigens to thymocytes (62). It has also been shown that self-specific B cells in the thymus can acquire antigens *via* B cell receptor- (BCR) mediated endocytosis and promote tolerance by presenting these antigens to thymocytes (64). Moreover, class-switched B cells may also play an important role in driving tolerance to unique B cell antigens and such tolerization of T cells would be crucial during the adaptive immune response in the periphery (15, 63, 65). Given that thymic B cells and their role in antigen presentation were reviewed at length (66), we will primarily discuss antigen distribution and presentation in mTECs and thymic DCs.

Taken together, the thymus is a unique place, where the vast majority of antigens derived from the host’s own tissues is presented to mediate clonal deletion or Treg conversion of self-reactive T cells. A large proportion of these antigens are directly presented to thymocytes by a unique population of AIRE^+^ mTEC^High^. Moreover, thymic populations of hematopoietic APCs also participate in central tolerance mechanisms by direct presentation of antigens that cannot be presented by mTECs, such as blood-borne antigens, antigens derived from microbiota, or B cell specific antigens.

## Indirect Presentation of Tissue-Restricted Antigens

As described in the previous chapter, mTECs are a critical cellular source of self-antigens in the thymus. However, the total number of mTECs is quite limited (approx. hundreds of thousands per thymus in a young mouse (67, 68)). Moreover, each individual mTEC presents a distinct set of TRAs that constitutes a mere fraction of this TRA pool (16). In addition, mTECs were recently found to be highly heterogeneous, comprised of cell subsets, some of which weakly displayed or were incapable of producing or presenting TRAs (38). Another frequently discussed issue is the fact that antigen processing and presentation often differs in mTECs and peripheral APCs, which begs the question of how closely mTEC-centered central tolerance mimics antigen presentation in the periphery, and thus ensures the scope and stringency of negative selection (69). This concept has led researchers to consider whether mTEC-autonomous production and presentation of self-antigens is sufficient to establish a fully operational immune tolerance or if such a task is beyond their collective capacity and discretion.

Nearly two decades ago, *Gallegos and Bevan* provided insight into this issue. They convincingly showed that thymic clonal deletion of OVA-specific CD4^+^, and to some extent CD8^+^ T cells, is dependent on antigen presentation by bone marrow-derived (BM) APCs. Since the expression of the membrane bound OVA (mOVA) antigen under RIP was directed exclusively to mTECs, the authors concluded that mOVA must be transferred to BM APCs and displayed in the context of their MHC molecules for the efficient deletion of cognate T cells (70). Given that developing T cells reside in the medulla for 4-5 days as they rapidly move to scan pMHCs on a variety of APCs (67, 71), such antigen transfer from mTECs to BM APCs can significantly reinforce the establishment of central tolerance. This new phenomenon which “sealed the gaps” in mTEC-driven tolerance was referred to as indirect antigen presentation (70).

The original data obtained with the RIP-mOVA model was complemented by another transgenic system, where OVA was produced only in those mTECs which expressed AIRE, e.g. Aire-OVA knock in (Aire-OVA-KI) mice (72). In sharp contrast to the *Gallegos and Bevan* study, where the deletion of mOVA-specific OT-II T cells was found to be completely dependent on the indirect presentation by BM APCs, direct presentation of OVA by mTECs was sufficient to delete OTII T cells in the Aire-OVA-KI model. The explanation for this discrepancy is likely due to the fact that in the RIP mOVA model, the mOVA is expressed predominantly by mTEC^Low^, whereas the expression of OVA in Aire-OVA-KI mice is restricted to the mTEC^High^ subset whose antigen presentation capacity is robust (72). Since mTEC^Low^ are poorly presenting APCs, their presentation of mOVA is presumably insufficient to induce a proper clonal deletion and/or deviation of OT-II T cells to Tregs without the support of DCs. This hypothesis is supported by the evidence from *Hinterberger et al.* that showed that a reduction in the expression of MHCII on mTECs leads to the impaired selection of OVA-specific T cells, regardless of DCs depletion (48). On the other hand, indirect presentation was repeatedly shown to be dependent on AIRE, since it upregulates the expression of several chemokines which attract DCs to the vicinity of AIRE-expressing mTECs (72–74). AIRE also supports indirect antigen presentation by suppressing CTLA-4 expression in mTECs, hence keeping the key costimulatory role of CD80/86 molecules on BM APCs for agonist selection of Tregs uncompromised (75). In fact, the deviation of T cells into Tregs was found to be dependent on AIRE in both modes of antigen presentation (72). This is consistent with the observation that for the agonist selection of Tregs, the presentation of TRAs by APCs residing in the medulla of the thymus is absolutely necessary (76) which is in contrast to the requirements for clonal deletion of T cells that appears to be much less dependent on a functional medullary microenvironment (74, 77, 78).

Using two-photon microscopy of *ex vivo* thymic slices from RIP mOVA mouse, it was recently shown that most of the mOVA specific CD8^+^ OT-I T cells were activated by BM APCs through indirect antigen presentation, while the activation of CD4^+^ OT-II T cells was found to be equally dependent on both direct and indirect mOVA presentation. In contrast to the RIP mOVA system, RIP OVA^HI^ mice which produced an intracellular form of OVA under RIP, showed a much higher activation of OT-II T cells by BM APCs than OT-I T cells (79). Thus, it seems that subcellular localization of OVA predicates its predominant indirect presentation on MHCI or MHCII molecules. Importantly, this study also suggested that when polyclonal T cell repertoire is considered, an indirect antigen presentation played a primary role in the deletion of CD4^+^ T cells. In addition, there was evidence that an indirect presentation was, in general, as crucial as the direct presentation of antigens by mTECs in both CD4^+^ and CD8^+^ T cell tolerance (79).

The indispensable role of indirect antigen presentation in the context of the polyclonal T cell repertoire was ascertained by TCRα sequencing of BM chimeras that exhibited partial or full MHCII deficiency on mTECs and BM APCs, respectively (8). It has been found that TCR specificities sensitive to indirect presentation generally do not overlap with those specificities engaging mTECs. Furthermore, BM APCs were found to be crucial not only for clonal deletion but for the generation of Tregs where approximately 30% of unique Treg TCR specificities were dependent on MHCII presentation by BM APCs. Moreover, a vast array of TCR sequences that were either deleted or deviated into Tregs by BM APCs turned out to be dependent on AIRE. Paradoxically, these T cell clones could not be deleted or transformed into Tregs by direct antigen presentation (8). A logical explanation for this observation relies on the fact that mTECs and BM APCs possess different antigen processing machinery that results in the presentation of distinct peptides from a particular TRA (80–82). Hence, indirect presentation not only raises the number of cells which present TRAs, it also extends the repertoire of T cell clones affected by the processes of central tolerance. In support of these results, other studies have reported a requirement for indirect presentation to delete or deviate into Tregs those T cells which engage certain AIRE-dependent TRAs, namely proteolipid protein (PLP) (35, 83), interphotoreceptor retinoid binding protein (IRBP) (33), or prostate-specific antigen MJ23 (9).

As previously stated, the routine use of scRNAseq by us and others has led to the exploration of thymic BM APC heterogeneity (39, 53). Even though it has been repeatedly shown that thymic DCs are those BM APCs which participate in indirect antigen presentation, their relative contribution to this process remains unclear (8, 9, 28, 79, 83–86). Importantly, all thymic DC subsets are capable of obtaining antigens from mTECs (53, 87). Nevertheless, while pDC, cDC2, and moDC are also known to present the antigens acquired from outside of the thymus (59–61), cDC1 and aDC seem to establish central tolerance primarily through indirect presentation of mTEC-derived antigens (8, 54, 88). Indeed, cDC1 and aDC are localized to the medulla in proximity to AIRE-expressing mTEC^High^ (39, 74). Moreover, the cooperation of cDC1 with mTECs was found to be indispensable for keeping the process of tolerance establishment operational, since autoimmune manifestations are much more profound in mice that are deficient in both cDC1 and mTECs compared to mice deficient only in the mTEC or cDC1 cell compartment (89). Although cDC1 deficient mice do not display differences in their overall frequency of Tregs compared to WT mice (89), their Treg repertoire was found to be aberrant, mainly in respect to those clones which recognized AIRE-dependent TRAs (8). Nevertheless, recent experiments with cDC1 deficient *Batf3* KO mice have shown that merely 2% of clonally deleted T cells and 12% of generated Tregs were completely dependent on the cDC1 lineage (88). Another report which used *Batf3* KO mice as a model, also showed a negligible role of cDC1 in clonal deletion of CD8^+^ T cells (90) which, given the robust cross-presentation capability of cDC1 (91), was surprising. On the other hand, thymic cDC2 revealed an efficient cross-presentation of mTEC-derived antigens to CD8^+^ T cells in *ex vivo* thymic slices (79), which indicated their contribution to the deletion of self-reactive cytotoxic T cells. It is of note that several studies have provided evidence that Tregs are generated by cDC2 and not by cDC1 (9, 86, 92). Notably, it was observed that a CCR7 deficient thymus displayed a reduction in the cDC1 lineage which lead to an enhanced Treg selection by cDC2 that expressed low levels of MHCII (92). However, this result is puzzling in the context of recently described CCR7^+^ aDC subsets which are marked by high MHCII expression with several transcriptomic analyses showing that these subsets are molecularly fully equipped for Treg generation (39, 53, 54). Moreover, a recently published study from our lab showed that moDC can enhance Treg generation in the thymus under inflammatory conditions *via* the acquisition of mTEC-derived antigens (53). Thus, at this juncture, while it seems that each thymic DC subset can contribute to indirect antigen presentation, the level of their contribution to recessive versus dominant tolerance in respect to the accompanying physiological circumstances requires further clarification.

**Figure 1:**
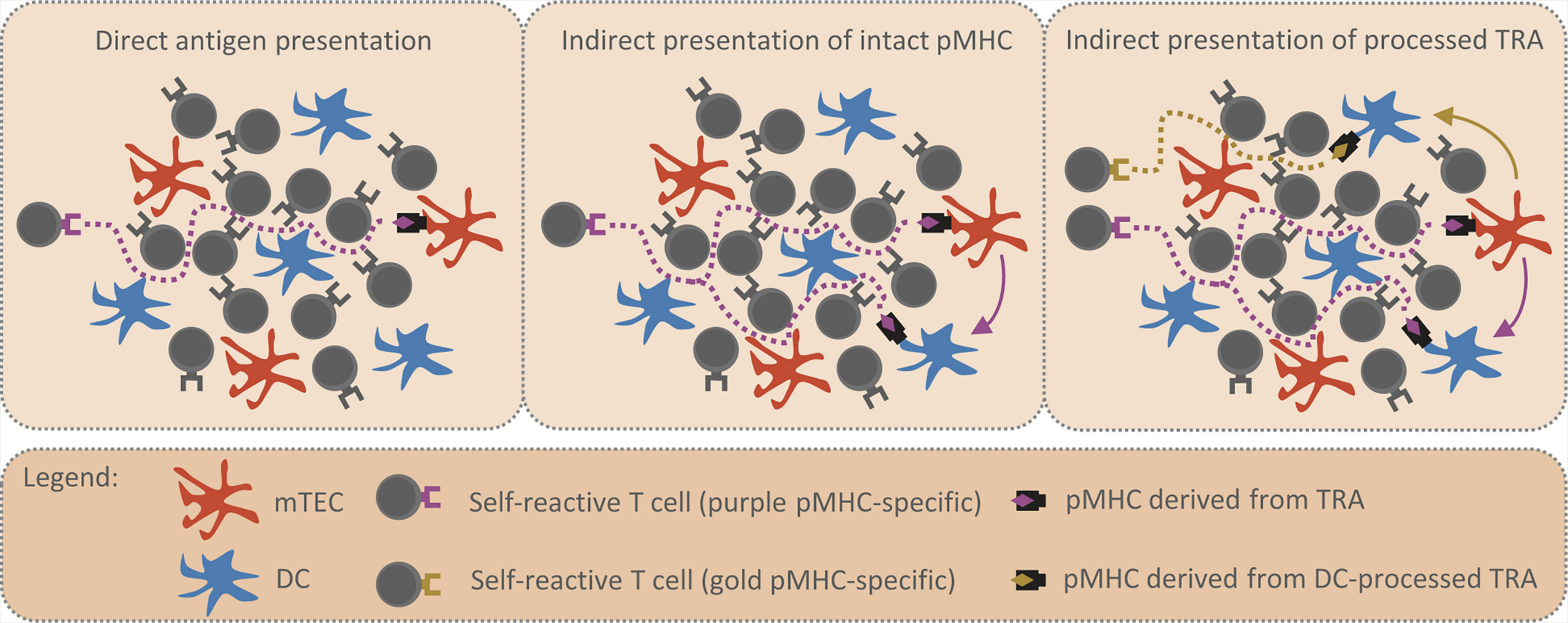
Reinforcement of central tolerance by indirect presentation. The schemes depict model situations in which self-reactive T cells (purple and golden TCR) migrate through the thymic medulla to engage their cognate TRA (purple and golden rhombus) presented by mTEC (in orange) or DC (in blue) and undergo the processes of central tolerance. Possible migration pathways of self-reactive T cells are visualized by the dotted lines. The first scheme (left panel) displays the situation where TRA is presented directly by a single mTEC, thus, there is a low probability that a self-reactive T cell will encounter the mTEC and be tolerized. In the second scheme (middle panel), the intact pMHC shown in the left scheme is transferred from mTEC to DC (purple arrow). The antigen presentation is enhanced, since the same TRA is presented both directly and indirectly by mTEC and DC, respectively. The third scheme (right panel) captures the situation in which the TRA is transferred to (golden arrow) and subsequently processed by DC. Since the antigen processing machinery of DC is distinct from that of mTEC, a DC-processed pMHC complex (golden rhombus) is recognized by a self-reactive T cell of different specificity (golden TCR) from the original (purple TCR). Therefore, indirect presentation not only enhances antigen presentation in the medulla (middle panel), it also broadens the repertoire of T cell clones subjected to processes of central tolerance (right panel).

It was shown recently that the abrogated phagocytic activity of BM APCs led to impaired deletion of CD8^+^ T cells (93). The authors of this study proposed a model that illustrated the clearance of self-reactive T cells by BM APCs preventing their escape from clonal deletion and subsequent autoimmune manifestations in immune periphery. This process was found to be dependent on the expression of phosphatidylserine and the scavenger receptor, TIM-4, on apoptotic cells and phagocytes, respectively. Thus, it seems that rapid phagocytosis, besides indirect antigen presentation, represents an essential capability of BM APCs to establish central tolerance. In fact, clonal deletion was found to be most efficient when T cells engaged indirectly presented antigen on the same BM APC which also phagocytosed such a T cell (93). However, based on the current knowledge, we propose that the observed breakdown of central tolerance in phagocytosis-deficient thymus is likely caused by deficiencies in indirect antigen presentation (83, 88) (see **Figure 1** highlighting physiological benefits of indirect antigen presentation).

## Cooperative Antigen Transfer

In 1994, *Bruno Kyewski´s* group reported that thymic DCs acquire antigens which are produced by thymic epithelium (94). This original finding gained importance ten years later when *Gallegos and Bevan* showed that indirect presentation of mTEC-derived antigens by thymic DCs is crucial for the maintenance of central tolerance (70). Hence, it became obvious that the transfer of antigens from mTECs to DCs, referred to as “Cooperative antigen transfer” (CAT) (69), is a prerequisite for indirect antigen presentation. According to our data and the results of others, all currently described DC subsets participate in CAT (53, 83, 87, 95). However, given that their heterogeneity is determined by a distinct gene expression profile (39, 53), each subset might employ a distinct mechanism to achieve it. Theoretically, CAT can be mediated by cell contact-independent and several cell contact-dependent mechanisms, namely via: i) exosomes, ii) trogocytosis, iii) gap junctions and iv) endocytosis/phagocytosis, and was shown that it involves antigens with nuclear, cytosolic or membrane localization (83). Regarding the cell contact-independent mechanism, it was reported that human mTECs secrete exosomes which contain TRAs when cultured *in vitro* (96). However, it has been repeatedly shown using transwell assays that exosomes do not serve as a source of TRAs. Indeed, CAT requires and is dependent on a cell-cell contact (88, 95, 97).

Trogocytosis is a process in which two cells exchange portions of their plasma membranes (98). For example basophils were shown to obtain intact pMHCII complexes from DCs through trogocytosis, and thereby served as APCs, even though they did not express antigen presenting machinery genes, including those encoding MHCII (99). By the same token, trogocytosis has been suggested to drive CAT of intact pMHCII molecules in the thymus ensuring their rapid presentation to T cells (83, 97). Paradoxically, while pMHCII molecules are localized to lipid rafts, these membrane microdomains were found to be dispensable for operational CAT (95). In our latest study, we took advantage of a Foxn1^Cre^Confetti^Brainbow2.1^ model in which we directly compared the transfer of membrane-bound CFP with cytosolic RFP or YFP from mTECs to DCs (87). Strikingly, the efficiency of the transfer of CFP was weak in comparison to cytosolic antigens. Additionally, in marked contrast with transfer of cytosolic antigens, the acquisition of CFP was negligible in all thymic DC subsets, except XCR1^+^ aDC. Hence, we focused on XCR1^+^ aDC and visualized the differences in their uptake of CFP, RFP, and YFP using imagestream. Notably, while RFP and YFP were strictly localized to the intracellular vesicles of XCR1^+^ aDC, CFP was localized in their plasma membranes. Hence, this result indicates that the mechanism of CAT in the context of cytosolic antigens differs from that of membrane-bound molecules and suggests that XCR1^+^ aDC utilize trogocytosis to perform CAT.

Since gap junctions manage to transport particles of molecular weight up to 1,8 kDa (100), transfer of small, cytosolic peptides might occur through this mechanism. Although all subsets of thymic DCs robustly acquire cytosolic, mTEC-derived antigens, XCR1^+^ aDC were shown to also excel in this mode of CAT (54, 87). As previously mentioned, cDC1 and XCR1^+^ aDC reside in permanent, close contact with AIRE^+^ mTECs (39, 74). Hypothetically, in this niche gap junctions might be formed between mTECs and DCs to drive CAT. However, so far there has not been published evidence to support this hypothesis.

AIRE^+^ mTECs exhibit a rapid turnover (101) and a tangible fraction matures into a senescent/apoptotic post-Aire mTEC (43). Although these mTEC subsets downregulate genes encoding antigen presenting machinery, according to a recent study, they retain high levels of TRA expression (38). Hence, apoptotic mTECs might serve as a reservoir of TRAs and act as an ideal phagocytosis substrate for DCs residing nearby. Indeed, experiments using a mouse strain which exhibit a knocked out scavenger receptor CD36 showed that cDC1 used this receptor to engulf apoptotic mTECs (88). An array of T cell clones whose clonal deletion/agonist selection relied on cDC1, also depended on functional CD36 and the lack of this receptor led to autoimmune manifestations. However, while CD36 seems critical for the establishment of central tolerance, cDC1 are endowed with yet another mechanism of CAT. A comprehensive study from *Bernard Malissen´s* lab unravelled the complex transcriptomic changes underpinning cDC1 homeostatic maturation into XCR1^+^ aDC in the thymus. Interestingly, this process resembles the immunogenic maturation of peripheral DCs or their maturation within tumors (54, 102). Notably, the maturation of cDC1 within tumors is driven by the scavenging of apoptotic tumor cells and is at least partially dependent on another scavenger receptor, AXL (102). To some extent, mTECs resemble tumor cells, since their DNA is highly stressed due to the AIRE-mediated formation of DNA double-strand breaks (103, 104). Thus, hypothetically, AXL-mediated CAT might drive cDC1 maturation in the thymus. There seems to be a consensus that thymic maturation of cDC2 converges with that of cDC1 into aDC phenotype (54, 55). Interestingly another scavenger receptor, TIM-4, is expressed by thymic cDC2 (53). Since the absence of TIM-4 abrogates the uptake of apoptotic bodies by thymic DCs and causes the breakdown of central tolerance (93), we posit that this molecule is also one of the drivers of CAT. Correspondingly, antigen uptake by thymic cDC2 or cDC1 was shown to be completely inhibited after the administration of Cytochalasin D and NH_4_Cl, inhibitors of phagocytosis (57).

With the exception of scavenger receptors expressed by DCs, chemokines and immune receptors expressed by mTECs are also considered to be critical molecular determinants of CAT. As mentioned in the previous chapter, mTECs express various chemokines in an AIRE-dependent manner which attract DCs of both cDC1 (XCL1) and cDC2 (CCL2, CCL8, CCL12) lineages to the vicinity of mTECs to facilitate CAT (73, 74, 105). In this context, we have recently shown that mTECs express Toll-like receptor (TLR) 9 whose signaling upregulates the expression of a set of AIRE-independent chemokines (53). This resulted in an enhanced migration of moDC to the thymic medulla, increased their potency for CAT, and in general, decreased the cellularity of thymic cDC1. Given that mice with the ablation of TLR9 signaling specifically in mTECs, displayed a decreased frequency and functionality of Tregs, it suggests that mTEC-produced chemokines which drive the enrichment of moDC in the medulla positively modulate agonist Treg selection. Recently, the checkpoint molecule, CTLA-4, expressed on the surface of mTECs was found to negatively affect the transfer of mTEC-derived MHCII molecules to cDC2 and more overtly to cDC1 (75). Since the silencing of CTLA-4 is AIRE-dependent event, AIRE also sustains CAT through this mechanism.

Finally, it has been postulated that adhesion molecules play a key role in CAT. Interestingly, thymic DCs exhibit a high expression of EPCAM, an adhesion molecule which is a standard epithelial cell marker (83, 95). Since it was observed in Foxn1eGFP knock-in mice, that those DCs which displayed a high positivity for mTEC-derived eGFP also possessed high levels of EPCAM, it was assumed that they acquired EPCAM along with eGFP from mTECs (83). Nevertheless, a recent study verified that EPCAM^+^ DCs express mRNA levels encoding this molecule comparably to mTECs, arguing that thymic DCs themselves produce EPCAM (95). Interestingly, thymic DCs outcompeted splenic DCs in their competence to perform CAT *in vitro* (83, 95). Since splenic DCs lack the expression of EPCAM (95), it is possible that the high expression of EPCAM by thymic DCs is a contributing factor to their efficient performance of CAT.

## Preferential Pairing in CAT

CAT has been described as a very complex process primarily because of the previously found heterogeneity of thymic APCs. Historically, CAT was shown as unidirectional process from mTECs to thymic APCs but specifically attributed to thymic DCs (53, 79, 83, 95). This unidirectionality advocates that CAT is a tightly regulated process (potential regulators were detailed above) that requires specific molecules to be expressed by both donors (TECs) and acceptors (thymic DCs), which also suggests that their differential expression affects the effectivity of CAT. This statement is supported by observations that distinct subtypes of DCs vary in their capacity to acquire TEC-derived antigens. Whereas CAT to cDC1 and cDC2 was reported to occur with the same efficiency, the transfer of antigens to pDC is fairly limited (53, 95). Notably, pDC were shown to be attracted to Hassall’s corpuscles, the structures formed by Post-Aire mTECs, which due to the lower expression of MHCII and persistent production of TRAs are considered as a source of self-antigens for CAT (47, 106). Accordingly, it was shown that the homing of thymic pDC into Hassal´s corpuscles in the human thymus endows pDC with the ability to generate Tregs (107). These findings suggest a role of thymic pDC in CAT specifically from Post-Aire mTECs. We have recently documented that the thymic moDC could also be drawn to the proximity of Post-AIRE^+^ mTECs due to the enhanced expression of pro-inflammatory chemokines (40, 53). This “preferential pairing” between specific subsets of TECs and thymic DCs has also been suggested by others. Notably, *Perry et al.* used as a model antigen GFP expressed only by AIRE^+^ mTECs in the thymus (Aire-GFP mouse model). The BM chimeras of WT cells injected into the Aire-GFP mouse revealed the antigen transfer of GFP specifically to XCR1^+^ DCs and only limited transfer to SIRPα^+^ cDC2 (88). On the other hand, the OVA antigen from the RIP-mOVA mouse model, whose expression is enriched in mTEC^Low^ or Post-Aire mTECs was transferred to SIRPα^+^ cDC2 with higher efficiency than to cDC1 (72, 79). These observations let us to predict that distinct subsets of thymic DCs acquire antigens from distinct subsets of TECs.

Our recent publication aimed to test this prediction by using several *Cre* reporter mouse models in which the expression of fluorescent TdTOMATO (TdTOM) protein is enriched in different subsets of TECs (87). The crossing of previously characterized *Cre*-based models with *Rosa26^TdTOMATO^* led to the generation of Foxn1^Cre^Rosa26^TdTOMATO^ where TdTOM is expressed by all TECs (53, 108), Csnb^Cre^Rosa26^TdTOMATO^ that restricts its expression to mTEC^High^ and their close progeny (36, 109), and Defa6^iCre^Rosa26^TdTOMATO^ where TdTOM mimics the expression of AIRE-dependent TRA while its production is limited to a minority of AIRE^+^ mTEC^High^ and their progeny (110). Using linear regression correlations with the predominant expression of TdTOM in a certain population of TECs and TdTOM transfer to distinct subsets of thymic DCs, the study demonstrated that CAT is mediated predominantly by preferential pairing between mTEC^Low^ and cDC2, mTEC^High^ and XCR1^+^ and XCR1^-^ aDC, and Post-Aire mTECs and pDC. Interestingly, two populations of thymic DCs, XCR1^+^ cDC1 and moDC did not reveal any or showed a limited correlation despite their high participation in the process of CAT (87). Since the previously mentioned study from Perry et al. described the XCR1^+^ DCs as the only DC-subtype which was able to acquire the GFP antigen from mTEC^High^, one can assume that this transfer was directed to mature XCR1^+^ aDC (88). On the other hand, as previously pointed out, because the XCR1^+^ aDC subset was shown to descend from XCR1^+^ cDC1 and their maturation in tumor tissues was shown to be dependent on antigen uptake, antigens expressed by mTEC^High^ may be indeed preferentially acquired by cDC1, which then initiates their maturation to aDC (54, 102). To verify such a scenario, the future identification of regulators of CAT and their subsequent genetic ablation will be necessary to test the prediction that XCR1^+^ aDC should not be generated in the absence of CAT from mTEC^High^ to XCR1^+^ cDC1 (102).

Remarkably, the situation with the thymic moDC population seems to be quite different. Using Foxn1^Cre^Confetti^Brainbow2.1^ mice and mixed BM chimeras where fifty percent of DCs express TdTOM, we recently showed that thymic moDC represent the major subtype which is responsible for the acquisition of antigens from multiple TECs or other DC-subsets (87). This probably reflects their enhanced migration capacity and phagocytic activity (53). Also, the fact that antigens could be transferred from one thymic DC to another thymic DC, challenges the dogma of exclusively unidirectional antigen transfer from TECs to DCs. Thus, it is clear that self-antigens produced by mTECs could be shared and presented to developing thymocytes by many distinct thymic DC-subtypes (87).

**Figure 2:**
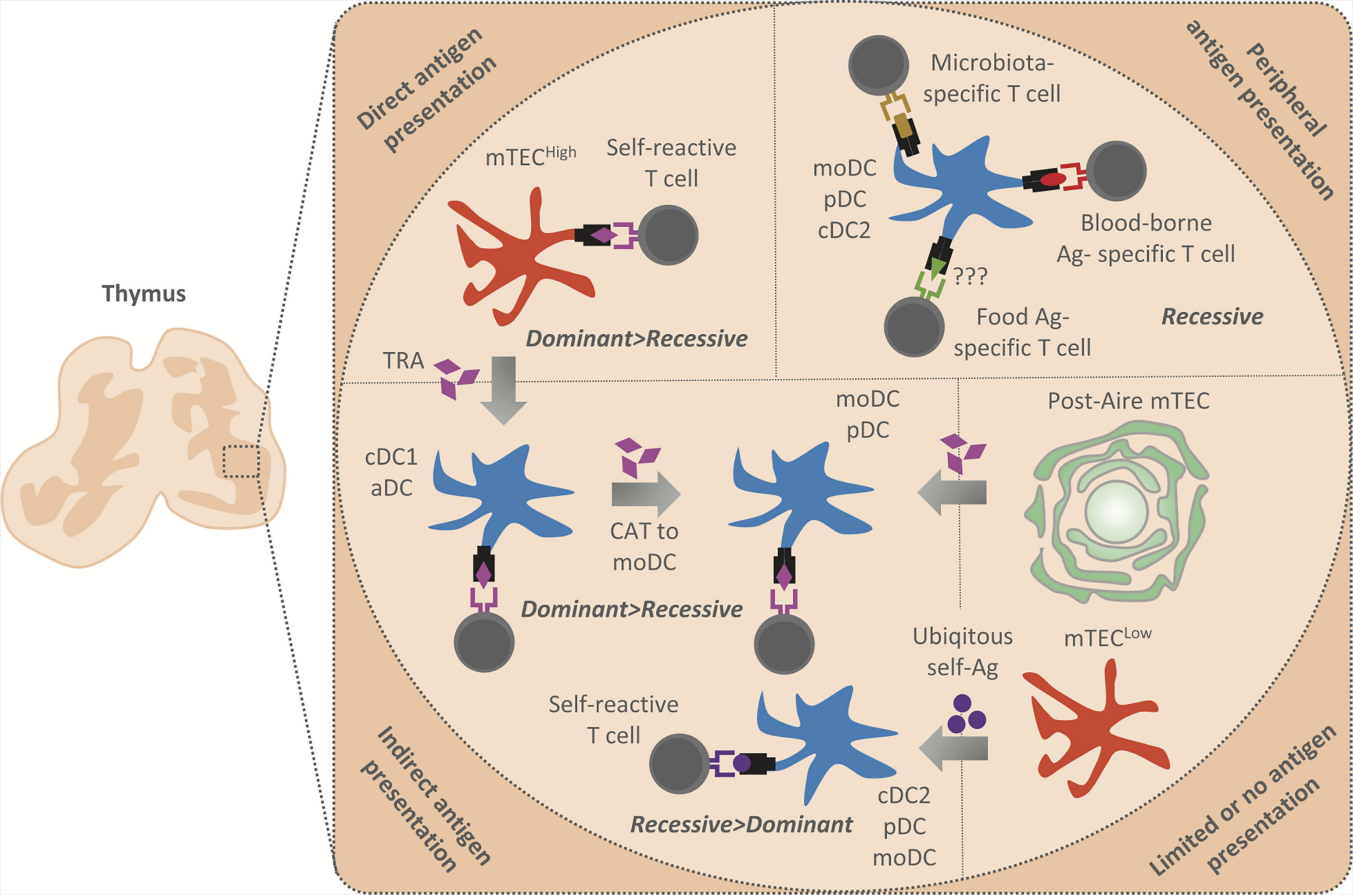
Summary of antigen presentation modes in the thymic medulla. The antigens presented in the thymic medulla are of both intra and extrathymic origin. Their presentation leads to the establishment of both dominant (Treg induction) and recessive (clonal deletion) tolerance. Peripheral antigens, i.e. blood-borne antigens (Ag) (in red), microbiota antigens (in gold), and possibly food antigens (in green) are delivered into the thymus by moDC, pDC or cDC2, and presented by these APCs to establish recessive tolerance. Note that blood-borne antigens are presented in the cortico-medullary junction where an extensive vasculature is situated. TRA (in purple) are generated by mTEC^High^ which either present them directly to establish dominant and recessive tolerance or transferred to cDC1 and aDC in their vicinity by CAT (gray arrows). cDC1 and aDC then establish recessive and more effectively dominant tolerance through indirect TRA presentation. Since moDC strongly acquire antigens from other DC, we suggest that these cells acquire TRA from cDC1 or aDC to enhance the establishment of dominant and recessive tolerance. Post-Aire mTEC which are part of Hassal´s corpuscles have limited antigen presentation capacity, however, maintain a high TRA expression. We suggest that Post-Aire mTEC serve as a reservoire of TRA for moDC and pDC, which seems to interact with them. Thus, TRA transfer from Post-Aire mTEC to moDC and pDC might lead to indirect presentation and establishment of both dominant and recessive tolerance. Finally, cDC2, pDC and moDC also acquire antigens from mTEC^Low^ which express a low amount of TRA and are limited in their antigen presenting capacity. Indirect presentation of antigens tranferred from the mTEC^Low^ subset is presumed to lead to the induction of recessive tolerance, since these antigens are ubiqitously expressed (in violet).

Having defined the main mechanistic framework of “preferential pairing” in CAT, the major question regarding the physiological consequences of this process in the central tolerance remain to be determined. As previously described, the CAT and subsequent indirect presentation of TEC-derived antigens to thymocytes help to overcome the recognizable limitations of mTEC-mediated tolerance, and thus extend the scope of self-antigen presentation in the thymus (6, 8, 48). As we have suggested, the recognition of ubiquitous antigen leads to T cell clonal deletion, whereas the recognition of TRA-like antigens generally promotes diversion to Treg lineage (14, 34). Thus, preferential pairing in CAT might underline the dichotomy of the selection process: either widening the scope of only Treg-generation or clonal deletion. This proposition is supported by the fact that XCR1^+^ DCs that preferentially acquire TRA-like antigens from mTEC^High^ are crucial for the generation of Tregs (8), whereas the SIRPα^+^ DC that acquire ubiquitous antigens from mTEC^Low^, cTECs or other DC-subsets are more attributed to clonal deletion (14, 111). Thus the “preferential pairing” in CAT between specific subtypes of TECs and DCs can be viewed as a crucial process in discriminating between clonal deletion and Tregs selection and also enabling the spreading of the antigens for both arms of central tolerance, recessive and dominant (see **Figure 2** summarizing modes of antigen presentation in the thymus).

## Conclusion

In the last decade, we have witnessed significant growth in our understanding of the contribution of mTEC- and DC-cell autonomous versus mTEC-to-DC cooperative presentation of self-antigens to selection processes which underline the establishment of central tolerance. However, the question of whether and how the individual subsets of mTECs and DCs provide a functionally non-redundant contribution to the deletion of self-reactive clones or their conversion to Tregs remains unresolved. The major technical hurdle in this process is the absence of suitable organismal reagents which would permit the ablation of antigen presentation function in phenotypically defined individual APC subsets present in a thymic microenvironment. The cellular architecture of the medulla which, to certain extent, is the result of the interplay between cytokines and chemokines which regulate the recruitment, differentiation, maturation, and apoptosis of participating cell subsets and guide cell-cell interactions, inevitably generate an important framework within which selection processes must be thoroughly considered and intensively studied. The question of how TCR-pMHC affinity-based selection events are modulated within such a dynamic microenvironment is largely unknown. While many questions remain to be answered in order to understand the intricacies of T cell selection processes, primarily those concerning CAT (see **Box 1**), one thing is becoming clear. As illustrated by the existence of preferential partnership between specific subsets of TECs and DCs for CAT, despite the increasing complexity of our understanding how central tolerance operates, it seems that this process is largely deterministic. Having this in mind, it is reasonable to assume that in future, we will be able to decipher the principles of T cell selection and in turn apply them to various clinical therapeutic interventions. Revealing the molecular determinants which control and modulate presentation of self-antigens will be next important step towards a unified view of how the universe of self-antigens and its cellular distribution in thymus is functionally coupled to the T cell selection process.

Box 1 **Questions to resolve.**

How the spatial architecture of the medulla and its key structural features support CAT in respect to the distribution of various DC subsets in this microenvironment?

What soluble and cellular factors in the thymic medulla influence apoptosis of mTECs and thymic DCs serving as a substrate for CAT?

How the preferential localization of each particlar DC subset in the microenvironment of medulla, its cell mobility, phagocytic activity, and chemotactic ability contribute to its capacity to participate in CAT?

What are the molecular determinants regulating the preferential pairing of mTEC and DC subsets?

## Author Contributions

JB, MV, and DF wrote and finalized the manuscript. All authors contributed to the article and approved the submitted version.

## Funding

This work was supported by Grant 20-30350S from The Grant Agency of the Czech Republic (GACR).

## Conflict of Interest

The authors declare that the research was conducted in the absence of any commercial or financial relationships that could be construed as a potential conflict of interest.

## Publisher’s Note

All claims expressed in this article are solely those of the authors and do not necessarily represent those of their affiliated organizations, or those of the publisher, the editors and the reviewers. Any product that may be evaluated in this article, or claim that may be made by its manufacturer, is not guaranteed or endorsed by the publisher.
